# ‘It's a Balancing Act’: A Qualitative Analysis of the Experiences of Caffeine Use in Individuals With Bipolar Disorder

**DOI:** 10.1111/bdi.70171

**Published:** 2026-09-30

**Authors:** Sammy Eden, Michaela Sibsey, Sarah Dean, Javier T. Gonzalez, Kim Wright

**Affiliations:** ^1^ University of Exeter Exeter UK; ^2^ University of Reading, Whiteknights Reading UK; ^3^ University of Exeter Medical School, St Luke's Campus Exeter UK; ^4^ Department for Health, Centre for Nutrition, Exercise and Metabolism University of Bath Bath UK; ^5^ Department of Psychology University of Exeter, Washington Singer Labs Exeter UK

**Keywords:** bipolar disorder, caffeine, depression, lifestyle behaviours, mania

## Abstract

**Introduction:**

Bipolar Disorder (BD) is a complex and often chronic mood disorder that can cause significant distress and disruption to everyday life. The condition often requires lifelong treatment and self‐management. Caffeine is a lifestyle behaviour that could be useful in self‐management alongside traditional treatments, but further research is needed to explore the relationship between this lifestyle behaviour and its potential significance on affective state.

**Methods:**

Individuals with BD (*N* = 20) took part in qualitative semi‐structured interviews, engaging in discussion about their personal experiences of caffeine consumption in relation to BD mood symptoms. Interviews were analysed using Interpretative Phenomenological Analysis (IPA).

**Results:**

Analysis revealed the superordinate theme ‘it's a balancing act’ was present in the majority of the transcripts, describing the caffeine‐bipolar relationship. This superordinate theme comprised themes specifically linked to lower mood states, ‘little helper’ and ‘nothing works’ and others linked to higher mood states ‘increased manic‐ness’ and ‘can't recall/concentrate’, in addition to themes around habitual caffeine consumption ‘habit/routine’ and planned use of caffeine ‘strategic routine’.

**Conclusion:**

The complexities of the caffeine‐bipolar relationship are highlighted. Participants indicated that caffeine requires a very delicate balancing act, having the potential to be both helpful and harmful. Findings emphasise the importance of increasing knowledge surrounding caffeine use for both patients with BD and clinical professionals.

## 1 Introduction

Caffeine is a popular dietary component consumed by 80% of individuals every day [1]. It is a psychostimulant well known for its energy and mood‐altering abilities [2, 3]. Whilst it is considered safe for the majority of individuals, there may be scope to suggest limiting and/or monitoring its use within certain populations [4]. In relation to Bipolar Disorder (BD), evidence is largely from case reports [5] which have focused on the potential harm of caffeine use during mania [6, 7, 8, 9, 10, 11]. There is little other research regarding the effect of caffeine within bipolar depression, with the exception of one case report suggesting excessive caffeine consumption may have resulted in the onset of a depressive episode and increased anxiety during low mood [12]. In the literature on unipolar depression, however, habitual caffeine consumption has been suggested to have some protective effects against depression [13, 14]. It is also thought that the mood and energy enhancing effects of caffeine could make it desirable for an individual experiencing an episode of depression [15].

Clinical guidelines give little in the way of specific recommendations regarding caffeine use for those with BD. The British Association for Psychopharmacology (BAP) recommends clinicians to check for excessive use of caffeine and to encourage reducing caffeine intake in the event of sleep disturbance and/or heightened anxiety [16]. Guidelines from the Canadian Network for Mood and Anxiety Treatments (CANMAT) and the International Society for Bipolar Disorders (ISBD) suggest minimising stimulants, including caffeine [17]. There is no mention of caffeine in American Psychological Association (APA) practice guidelines for the treatment of patients with BD [18]. The lack of guidance is perhaps surprising as there is a common belief that individuals with mental disorders consume more caffeine and are more likely to use it in a harmful way [6, 19], with high caffeine consumption having the potential to result in psychotic symptoms [20, 21]. The lack of research combined with the combination of contradictory findings and large reliance on case reports indicates a need for further research to be conducted in this population, particularly given caffeine is such an inexpensive, commonly used and socially acceptable drug yet may have potentially adverse consequences in certain circumstances [1, 2, 22].

There are also theoretical reasons to expect that caffeine could be significant in the exacerbation and management of BD mood episodes. The Behavioural Activation System (BAS) dysregulation theory [23, 24] proposes the BAS, a hypothetical, superordinate, biobehavioural system that organises behaviour towards gaining reward from the environment, is dysregulated and hypersensitive in those with bipolar, such that it is easily and excessively activated as a result of rewarding stimuli, resulting in (hypo)manic symptoms. (Hypo)manic symptoms promote further reward striving and stimulation seeking behaviour, exacerbating BAS activation further, and increasing symptoms in an escalating cycle. It has been hypothesised that physical activity, another lifestyle behaviour that can be physiologically activating, could form part of this upward spiral [25]. Caffeine also possesses rewarding and stimulating properties that may allow it to function in a similar activating manner.

The integrative cognitive model of BD also proposes an upward spiral of behaviour, activation and symptoms [26]; which can be exacerbated by the extreme, personal appraisals made by the individual of their activated internal state. Within this model a trigger event that results in energised or activated feelings is appraised as an opportunity or as a threat. If the former, the individual may engage in ‘ascent’ behaviours to capitalise on this opportunity and drive mood and activation up further; if the latter, they may engage in ‘descent’ behaviours to pull mood and activity down.

Theoretically caffeine has the potential to act as such a trigger because of its effects on internal state, and also as an ascent behaviour (through additional caffeine consumption) or a descent behaviour (through caffeine avoidance). Given the lack of research in this area the current study sought to explore individuals' experiences of caffeine use and mood within the context of BD. The aim was to gain insight and further our understanding of individuals' views on the relationships that may exist between caffeine and BD mood symptoms. This is not about the separate experience of caffeine and mood, but individuals' perspectives on the influence of one on the other.

## 2 Materials & Methods

### 2.1 Participants

Purposive sampling was used to recruit 20 individuals with a research diagnosis of BD. This was to allow for variation in mood state and breadth of experiences for superordinate themes, whilst still allowing us to focus on specific examples at the idiographic level. Participants were recruited via advertisements in the local community and via national advertisement through voluntary third sector organisations (i.e., Bipolar UK). Participants had to meet the following inclusion criteria: age 18 years or above, sufficient competency in English to understand and complete study measures and the interview process, a research diagnosis of BD I or BD II according to DSM‐V [27], and no current substance use disorder in the last 3 months as per DSM‐V. Additionally, individuals were required to consume some dietary caffeine during an average week. Participants lacking capacity, with high risk of suicide, recent, frequent, significant self‐harming behaviour, or current positive symptoms of psychosis as per DSM‐V were not included in the study.

### 2.2 Measures

All participants were assessed using the Structured Clinical Interview for DSM‐V Disorders (SCID) [27]; to ensure they met study inclusion criteria. Participants also completed a four‐item questionnaire adapted from the Caffeine Food Frequency Questionnaire [28] and a Caffeine Consumption Diary [29] to estimate habitual caffeine consumption on an average week over the past month. Participants were asked to note the number of days they had consumed coffee, tea, chocolate and soft drinks, sports drinks, or energy drinks and the quantity consumed each day.

The semi‐structured interview guide was developed by the research team. The interview guide was modified and restructured following consultation from qualitative experts external to the project team and another individual with personal experience of BD. Opening questions asked participants about their experiences of BD mood symptoms to ‘set the scene’ so the interview questions that followed could be tailored to their personal experiences. Individuals were then asked about their caffeine behaviours in isolation and then in relation to their BD mood symptoms with accompanying follow‐up questions. The interview schedule also included questions about experiences of physical activity; analyses pertaining to these questions are not reported here.

### 2.3 Procedure

The study received approval from the UK Health Research Authority (ref: 17/SW/0258). Participants verbally consented to participating in an initial introductory phone call, where a brief overview of the study was given. Interested participants giving written informed consent then completed an eligibility assessment by telephone which included the SCID‐V interview as well as additional questions to determine eligibility. Those meeting inclusion criteria and wishing to continue then completed the semi‐structured interview at a second appointment. All interviews were conducted via phone and lasted approximately 90 min.

### 2.4 Analysis

The transcribed semi‐structured interviews were analysed using Interpretative Phenomenological Analysis (IPA). Researchers specifically selected IPA as this analytical approach is committed to understanding participants' lived personal experiences [30, 31]. An inductive idiographic approach was taken, working from the bottom up, whereby themes and relationships between themes emerge directly using the participant voice and interpretations of their unique personal experiences to draw together common emergent themes [31]. Due to the relatively large sample, we employed a common themes approach to IPA focusing on key emergent themes across the group instead of detailed reporting of single case stories. The themes are commonalities of the group's subjective experiences, using the participant voice, and illustrated with specific individual examples [31].

Data analysis was conducted independently by two researchers (SE and MS), following the IPA process set out by Smith and colleagues [31]. This began with open coding, whereby both researchers independently noted initial ideas for each transcript [32]. Analysis was undertaken at the individual level, whereby the researchers examined the transcripts line by line, coding and interpreting the experiences and understandings of each participant, attempting to make sense of these [30, 31]. Emergent patterns and themes were subsequently interpreted and identified in participant responses to the questions, alongside potential explanatory inferences. This was performed first in five single cases and then across multiple cases within this set of five. This process was repeated four times ensuring all 20 transcripts received an in‐depth individual analysis before cross‐case analysis occurred. Researchers independently looked for patterns, emergent themes and potential explanatory inferences and then met regularly to discuss and review these. A diagrammatic structure was developed alongside participant narratives to illustrate the relationship between the emergent themes (Figure 1). Emerging themes were then cross‐referenced back to original transcripts for verification for a final time; the diagrammatic structure was modified as required, and any final discrepancies were discussed. The final themes, diagrammatic structure and narratives written were quality checked by a third member of the research team (K.W.) before undergoing audit and credibility checking by a fourth researcher (SD) who was independent from the data collection process. Descriptive statistics for quantitative data were produced using SPSS (Version 28, 2021).

**FIGURE 1:**
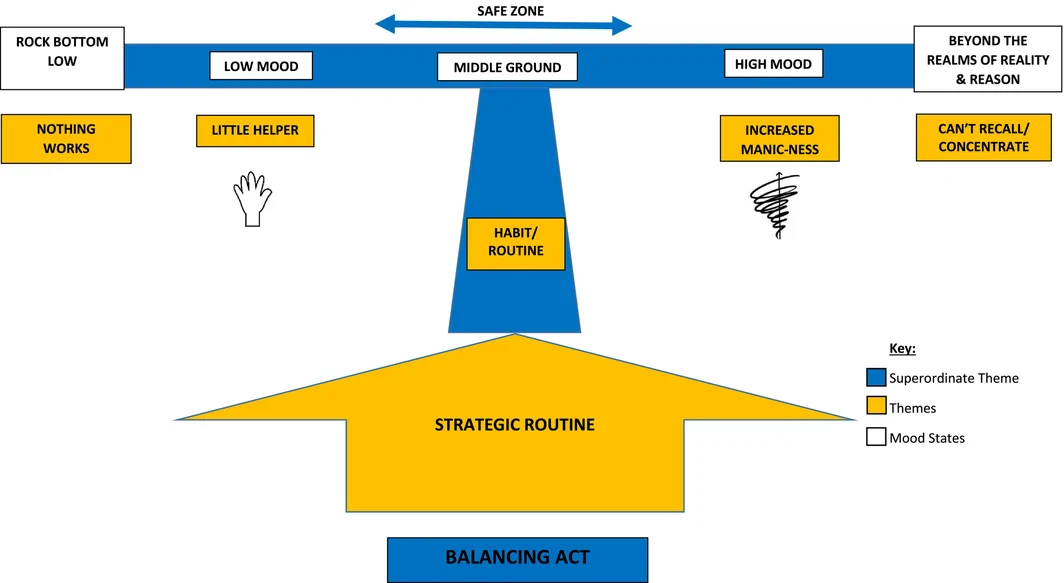
A diagrammatic structure representative of the themes and mood states arising from the caffeine‐bipolar relationship.

## 3 Results

### 3.1 Description of the Sample

A total of 20 participants with BD were interviewed, of whom 15 met research diagnostic criteria for BD I and five for BD II. This sample size allowed for variation in mood state and breadth of experiences for superordinate themes, whilst still allowing the focus to be on specific examples at the idiographic level. Nineteen participants were currently prescribed psychiatric medication. Participants were aged 33 to 71 (mean age of 49.2 ± 9.7); eight of the 20 participants were male, with the others being female. Fifteen participants described their ethnicity as white British, four as other white backgrounds and one as mixed multiple ethnic backgrounds. In reporting findings, participants were given pseudonyms to protect their identity; these are listed in Table 1 alongside caffeine habits. Coffee was the most commonly consumed caffeinated source: 19 participants reported they consumed coffee at least once in an average week. In addition, 18 reported they consumed tea, 16 consumed chocolate and 10 consumed caffeinated sport/soft/energy drinks. Half of the participants interviewed exceeded the recommended daily caffeine intake of 400 mg per day [33].

**TABLE 1:** A description of participants' caffeine behaviours.

Pseudonym	Caffeine types consumed over average week	Average daily caffeine consumption within/exceeding recommend daily 400 mg limit
Eric	Coffee, tea	Exceeding
Daniel	Coffee, tea, chocolate, sport/soft/energy drinks	Exceeding
Ester	Coffee, tea, chocolate, sport/soft/energy drinks	Exceeding
Gemma	Coffee, tea	Exceeding
Karen	Coffee, tea, chocolate, sport/soft/energy drinks	Exceeding
Penny	Coffee, tea	Exceeding
Owen	Coffee, tea, chocolate	Exceeding
Elizabeth	Coffee, tea chocolate, drinks	Exceeding
Katherine	Coffee, chocolate, drinks	Exceeding
Ryan	Coffee, tea, chocolate	Exceeding
Luke	Coffee, tea, chocolate	Within
Emma	Coffee, tea, chocolate, sport/soft/energy drinks	Within
Hannah	Coffee, tea, chocolate, drinks	Within
Victoria	Tea, chocolate, sport/soft/energy drinks	Within
Alexander	Coffee, tea, chocolate	Within
Jack	Coffee, tea, chocolate	Within
Anna	Coffee, tea, chocolate, sport/soft/energy drinks	Within
Katrina	Coffee, chocolate, sport/soft/energy drinks	Within
Sarah	Coffee, tea, chocolate	Within
Andrew	Coffee, tea, chocolate	Within

### 3.2 Overview of Themes

We identified a superordinate theme named ‘the balancing act’. Within this there were six higher order themes and seven subthemes. Higher order themes included ‘nothing works’, ‘little helper’, ‘increased manic‐ness’, ‘can't recall/concentrate’, ‘habit/routine’ and ‘strategic routine’. Within the theme ‘little helper’ was the subthemes ‘crutch’ and ‘comforting’ and within the theme ‘increased manic‐ness’ there was ‘the reverse of helpful’ and ‘complements mania’ subthemes. The theme ‘strategic routine’ encompassed three subthemes: ‘more caffeine’, ‘self‐care’ and ‘restriction of caffeine’.

#### 3.2.1 Superordinate Theme ‘the Balancing Act’

In total 18 of the 20 participants contributed to this superordinate theme which centred around the BD mood scale depicted within Figure 1. This depiction is based on the concept of traditional apothecary scales, which require a delicate balancing act between weights in the pans at either end for the beam for equipoise. The BD mood scale ranges from very low mood: ‘rock bottom low’ to very high mood: ‘beyond the realms of reality and reason’. In the centre is ‘middle ground’: a more stable mood, where participants are neither low nor high. The diagram also depicts a ‘safe zone’, whereby participants described having a ‘caffeine quota’ or ‘caffeine allowance’ which they would not exceed to keep their mood stable and closer to ‘middle ground’. The ‘balancing act’ theme was present within the transcripts in three ways. Firstly, participants actively described an overall ‘balancing act’, carefully monitoring lifestyle behaviours and their impact on BD mood symptoms, that was vital to manage their condition. Secondly, participants described actively being aware of the effects caffeine had in both lower and higher mood states and using caffeine strategically in the management of these mood states and symptoms. Finally, some participants did not explicitly describe their caffeine use as a ‘balancing act’ but nevertheless gave the sense that their caffeine consumption functioned in this way through their descriptions of their caffeine use. Refer to Supporting Information Table S1 for further insight into the balancing act superordinate theme.

#### 3.2.2 Lower Mood: Higher Order Themes ‘Little Helper’ and ‘Nothing Works’

Participants described caffeine having beneficial effects during low mood. One participant described caffeine as a ‘*little helper*’ [Penny] in relation to low mood which is where the higher order theme emerged from. This is represented in Figure 1 by the image of a helping hand. Caffeine was described as a ‘little helper’ in two ways: as a ‘crutch’ (subtheme), supporting daily functioning in periods of low mood, *‘It does give me that little tiny lift sometimes for me to do, you know, what I need to do just to survive’* [*Katrina*], a ‘comfort’ (subtheme), providing a sense of calm, reassurance and normality: *‘I find it quite comforting’* [Ester], *‘like a friend that sits alongside you as you're going through working it out’* [Gemma]. However, caffeine was not described as having the same beneficial, mood enhancing effects during episodes of extremely low mood, described by participants as ‘rock bottom low’, portrayed in the higher order theme ‘*nothing works*’; *‘I feel like it's not done anything’* [Hannah]. Caffeine was reported not to help with managing mood or improving symptoms of depression; *‘if my mood is really really low, it's very difficult to really improve it really’* [Penny]. Caffeine fulfilled two apparently opposing functions in low mood, seemingly distinguished by the intensity of the low mood. Interestingly seven participants reported experiencing both of these two different functions of caffeine, whilst two participants had no support for and shifted past the ‘little helper’ theme, only experiencing ‘nothing works’ in relation to very low mood. Refer to Supporting Information Table S2 for a breakdown of themes and subthemes for caffeine in relation to lower mood.

### 3.3 Higher Mood: Higher Order Themes ‘Increased Manic‐Ness’ and ‘Can't Recall/Concentrate’

In relation to higher mood, participants described how caffeine ‘increased manic‐ness’ (theme), exacerbating high mood and manic symptoms. This resulted in two possible outcomes and associated subthemes. The most common outcome was that this increased manic‐ness was the *‘reverse of helpful’* [Andrew]. Participants contributing to this ‘reverse of helpful’ subtheme described caffeine exacerbated manic symptoms in an unfavourable, detrimental or harmful manner. Victoria described *‘I've already got a lot of energy and, I'm wired, and my thoughts are racing, to add caffeine into that, well a lot of caffeine, it just, it doesn't sort of help. It makes everything worse’.* Conversely, caffeine's ability to increase manic‐ness *was* also described not to be unhelpful or harmful, as described by the subtheme ‘*complements mania*’ [Eric]. Participants described how they *‘drunk more coffee because I felt I need it to fuel my state of mania’* [Owen]; how it would *‘promote the mood’* [Andrew], *‘add to things being good’* [Ester], and allowed for greater focu*s*: *‘drinking coffee it will make me… more goal‐oriented and it makes me, you know, quite focused’* [Katrina], *fuelling productivity in a beneficial manner; ‘…I would get even more done. I would do it faster’* [Andrew]. Some participants described experiencing only one of these seemingly contradictory outcomes, whilst others described experiencing both. The ‘increased manic‐ness’ higher order theme is represented as an upward spiral, whereby high mood increases desirability of caffeine, *‘Going into a hypo or a manic phase… the coffee becomes much more attractive’* [Eric], resulting in increased caffeine consumption that exacerbates manic symptoms. This leads to an interactive effect that could potentially tip the individual over the edge from ‘high mood’ to very high mood, described as ‘beyond the realms of reality and reason’. ‘*I was already feeling*, *you know*, *quite revved up. It* [having a cup of coffee] *just tipped me right over the edge to the point where it was very hard to control my behaviour*.’ [Katrina].

At the right end of the scale in Figure 1 in relation to very extreme high mood episodes the higher order theme ‘can't recall/concentrate’ is depicted whereby participants commonly described being unable to recall caffeine consumed and/or the effects it had during extremely high mood. *‘It's just difficult to remember. I think if it was the opposite state you could remember more but it's difficult to remember things sort of being manic.’* [Emma]. Additionally, some participants described being unable to concentrate for long enough to make a caffeinated beverage; ‘*when I was really quite high and the way my brain was functioning wouldn't allow me to concentrate long enough to make a cup of coffee’* [Katherine] or consume it *‘I might not actually end up drinking the tea. It might end up going cold because I'm distracted’* [Elizabeth]. Refer to Supporting Information Table S3 for a breakdown of the ‘increased manic‐ness’ and ‘can't recall/concentrate’ themes and subthemes.

### 3.4 Higher Order Themes ‘Habit/Routine’ and ‘Strategic Routine’

Participants commonly described their caffeine consumption as a habitual routine: ‘*I tend to stick to the same pattern’* [Alexander] which is where the theme ‘habit/routine’ emerged. Participants described their caffeine routine as important, *‘it's routine… I have a really big thing about having routine with things, so I have a pattern I follow, where there is always the same thing happening, and that goes with coffee’* [Luke], helping their mood remain in ‘middle ground’, *‘The routine makes me feel more in control.’* [Anna]. In addition, participants also described what we have termed a ‘strategic routine’; a theme which denotes intentionally engaging in specific caffeine‐use behaviours seen as important to manage mood and bipolar mood symptoms. The apparent importance of having a strategic routine for caffeine use is reflected by the large arrow that is shown as underpinning and supporting the balancing arm mood scale (see Figure 1).

The ‘strategic routine’ higher order theme is further broken down into three subthemes. First, the subtheme ‘more caffeine’ emerged as participants described deliberately increasing caffeine consumption during periods of low mood to help them function and/or for the comforting effects caffeine provides; *‘I do think that if you're bipolar going into depression then strategic use of caffeine could be useful to give you a bit of a boost, a bit of a kick then’* [Andrew]. Second, the subtheme ‘self‐care’ emerged as participants described deliberately using caffeine as a way of taking care of themselves; *‘a little bit of self‐care’* [Elizabeth]. Participants described the mindful effects of caffeine, providing time to reflect *‘five minutes of just thinking things over, you need that thought process and just to have a think about your life’* [Hannah], and a sense of calm *‘It's a very relaxed homely smell, you know, it makes me calm… It instantly has like a reaction on my breathing because when I smell the coffee it's almost immediately triggers deep breath sensations… a relaxing breath.’* [Andrew]. Several participants commented that caffeine had a medicinal effect *‘it is to me medicine, it makes me feel better’* [Karen]. Participants also specifically described using caffeine as a form of self‐medication to counteract the side effects of their psychiatric medications (i.e., drowsiness). Finally, participants described ‘restriction of caffeine’ as part of their ‘strategic routine’ (the final subtheme) where they deliberately restricted caffeine intake or certain caffeine sources. Within this subtheme, some participants described switching to lower caffeine alternatives (e.g., tea instead of coffee) or decaffeinated alternatives (e.g., herbal teas), particularly during higher mood states; *‘on a higher end of the scale, I tend towards more herbal teas because I don't need the caffeine…’* [Elizabeth]. Participants also described restricting caffeine to a daily ‘quota’ or a certain timepoints to protect their sleep schedule; another lifestyle behaviour described as vital in the ‘balancing act’; *‘I do not like to overdo it because it will affect almost definitely my sleep pattern later on, you know going to sleep for Bipolar it's literally like the life and death importance’* [Penny]. Thus, planned use of caffeine by participants went beyond a simple routine or set of habits, instead representing often deliberate and carefully constructed patterns of use, flexible in response to mood state, and intended to maintain balanced mood. Refer to Supporting Information Table S4 for a breakdown of the ‘habit/routine’ and ‘strategic routine’ themes and subthemes. Sleep and physical activity also emerged as themes to be an important part of balancing act; these are further described in the Supporting Information, with a breakdown of themes provided in the Supporting Information Table S5.

## 4 Discussion

We identified a superordinate theme the ‘balancing act’ which was described by the majority of participants, whereby caffeine consumption and the effects of caffeine varied according to their bipolar mood state. In recognition of this participants described acknowledging their position on the BD mood scale at any given time, and proactively and reactively modifying caffeine consumption to self‐manage BD mood symptoms. A pivotal point of the balancing act for many appeared to be the adoption of a proactive strategic routine around caffeine use, involving deliberate behaviours such as consuming more caffeine, self‐care and the restriction of caffeine to help manage mood and BD symptoms. The balancing act is centred around the premise that caffeine was not described by participants to be universally helpful or harmful; this differed both between participants and within participants depending on mood state.

Our findings regarding the use of caffeine as a self‐management tool add to existing self‐management of BD literature. Participants in the current study recognised the importance of understanding and monitoring their caffeine behaviours to be an important part of this self‐management. Within the treatment of BD, this is exemplified by the success of group psychoeducation, which is largely focused upon teaching self‐management strategies in reducing the risk of BD relapse [34]. Research supports the importance of other, similar balancing acts in the self‐management of BD. Specifically, a previous qualitative study of self‐management of BD found participants described using multiple lifestyle behaviours to manage mood, including monitoring and restricting the use of mood‐altering substances [35], it is plausible caffeine could be included. By focusing on caffeine specifically in this study, we were able to hear about the nuances of caffeine use within the lives of these individuals.

The self‐management of BD is reflected within subdivisions of the balancing act superordinate theme. The strategic routine theme describes how participants deliberately and proactively employed caffeine use behaviours as tools to regulate affect that were important in the management of their mood and BD symptoms. We conceptualised strategic routine as not simply a habit‐based routine, but behaviours deliberately selected for regular use because they allow proactive regulation of mood. Specifically in relation to lower mood, participants described selecting to consume more caffeine to help them function, not merely because of its momentary comforting or mood elevating effects. Similarly, in periods of higher mood, many participants described deliberately restricting their caffeine intake to prevent exacerbating manic symptoms.

The strategic routine described by participants links closely to Gross' [36] model of emotion regulation. According to the model, the early stages of emotion regulation involve proactive strategies (i.e., before the emotion) such as situation selection, which refers to deliberately approaching or avoiding behaviours to regulate emotions at the earliest period of time, and situation modification which refers to actively attempting to alter the situation in attempt to change its emotional influence. In keeping with the ideas proposed in Gross' model, the strategic regulation of caffeine described by participants suggests they are specifically selecting or modifying the caffeine they consume, proactively using caffeine consumption as a tool to regulate their affect. Specifically in relation to lower mood, participants described strategically selecting to consume more caffeine in periods of lower mood to help them function or because of the comforting effects caffeine provided, whilst deliberately restricting their caffeine intake or certain sources of caffeine in periods of higher mood as to not ‘increase manic‐ness’ further and exacerbate manic symptoms.

Alongside strategic use, there were descriptions of caffeine being used reactively in both high and low states. Caffeine was described by participants as being more desirable during high mood; the desirable and stimulatory effects of consuming caffeine mean it could be considered an impulsive, pleasure‐seeking behaviour. There is evidence within the literature that individuals with BD are attracted to impulsive, stimulating, pleasure‐seeking behaviours in comparison to healthy controls [37]. Increased impulsivity is a common symptom in mania [38] with individuals likely to excessively engage in pleasurable activities that have a high potential for adverse outcomes [39]. Although the literature in relation to caffeine use is limited, previous research has found sensation seeking behaviours such as alcohol consumption to be associated with episodes of mania [40], with individuals being more likely to crave alcohol in higher mood states [41]. It is therefore both logical and possible that this may also be the case for caffeine, a drug which results in pleasurable effects. Although unlike alcohol which is considered a CNS depressant [42], caffeine is a central nervous system stimulant [43], raising the question of whether it may directly contribute to overstimulation and symptom escalation. Correspondingly, the majority of participants described that caffeine could have this effect. Although empirical research on the caffeine‐bipolar relationship is extremely limited [5], similarly to the current study, case study research has also highlighted that caffeine could be harmful in relation to higher mood, exacerbating high mood [6, 7, 8, 9, 10, 11].

As well as the possibility of caffeine having a direct effect on mania symptoms, there is also the potential for this to be mediated by cognitive appraisals of the internal state induced by caffeine. Within the integrative cognitive model of mood swings [26], our results confirm caffeine could be a ‘trigger event’ that leads to a change in internal state: participants described how consuming caffeine changed their mood and energy. Depending on their appraisal, this could lead to ascent or descent behaviours which, according to the theory, go on to exacerbate or reduce symptoms further. Caffeine consumption may also function as an ascent behaviour in and of itself, as some participants described using caffeine during high mood states because of its complementary effects. Similarly, during lower mood, consuming caffeine appeared to function as a beneficial ascent behaviour in that it elevated mood. However, it is important to note the limit of this relationship, as caffeine was not described as a helpful ascent behaviour during periods of extreme low mood (rock bottom low).

The description of caffeine in relation to high mood is also in keeping with the BAS dysregulation theory [44, 45, 46]. In this model, positive affect can represent an output of the BAS when activated. BAS activation is associated with increased energy, motivation and reward‐seeking behaviours. This appears to make stimulating behaviours such as caffeine consumption more attractive, resulting in impulse reward‐driven behaviours which only seek to activate the BAS further. This hypersensitivity increases both (hypo)manic symptoms and further engagement in caffeine consumption. Conversely, lower mood states can reflect underactivity of the BAS. When the BAS is under‐activated, this is associated with reduced energy, motivation and reward‐seeking behaviours. Consequently, stimulating behaviours such as caffeine consumption appear to become far less attractive, in keeping with the theory. The lack of engagement in these behaviours may seek to deactivate the BAS further, leading to reductions in approach behaviour and a downward spiral [47].

With regard to the sequences of events hypothesised above, it is important to acknowledge that no participant fully describes this upward spiral cycle from beginning to end. We therefore acknowledge that we are extending our understanding and interpretation of the data beyond the individual participants' descriptions, collating a possible sequence from across multiple participants. The current study has several other limitations. For one, the presence of other lifestyle behaviours, for example sleep, physical activity, and other dietary behaviours could have equal or greater importance than caffeine with respect to affect and BD mood symptoms. A detailed participant history was not gathered prior to the interview being conducted to reduce the burden placed on participants. As a result, limited background information was gathered and so we were unable to characterise the sample in terms of past and present treatment and classify participants according to their affective state when the interview was conducted. Finally, the research team members involved in data collection and analysis all specialised in health or clinical psychology. Although this could be considered a strength because it is psychologists who support people with BD to be able to self‐manage their condition, it could also be a weakness as phenomena have not been examined from other perspectives, for example considering the sociocultural impacts of caffeine use.

As the first qualitative study directly examining this area, the current study provides an important insight and understanding of the caffeine‐affect in this population. As BD mood state seems to be associated with caffeine use in this population, further research is warranted to better understand the relationship between both caffeine and bipolar symptoms, including any directional causality, and the conditions under which caffeine use might be helpful versus harmful.

Prospective, observational studies using experience sampling would allow hypotheses about the relationship between caffeine and symptoms to be tested within and between individuals, across time, within a free‐living environment. Studies in which caffeine dose is manipulated in individuals with bipolar disorder could help establish the causal effects of caffeine on symptoms but would require careful attention to the ethical and safety implications of such research. As BD mood state seems to be strongly connected to caffeine in this population, further research is warranted to better understand the relationship between both caffeine and affective states for individuals with this condition, including any directional causality and the conditions under which caffeine use might be helpful versus harmful. The current study highlights the complexities of the caffeine‐bipolar relationship, in that caffeine is likely to be both helpful and harmful depending on mood state and the effects are likely to be different for everyone.

As such it has practical implications for both individuals with BD and healthcare professionals in relation to self‐management and psychoeducation. In terms of self‐management, it would seem important that healthcare professionals speak to their patients about their caffeine use, encouraging the patient to reflect on how these behaviours may change according to their mood state and if and when they may be helpful or harmful. Discussing and evaluating lifestyle behaviours may have beneficial outcomes for both the patient and the healthcare system, as lifestyle changes are likely to be cost‐effective with probable positive effects on aspects of both physical and mental health, improving quality of life [47, 48]. This approach aims to help the individual to feel more in control of their wellbeing, learning about themselves and experimenting with changes to these lifestyle behaviours that will assist with self‐management [49]. Furthermore, caffeine could be enquired about as part of routine assessments, alongside other lifestyle behaviours such as smoking, alcohol and physical activity, which are already often routinely assessed.

## 5 Conclusion

The current study provides an initial stepping‐stone to understanding the caffeine‐bipolar relationship. Individuals described how caffeine required a delicate ‘balancing act’, having the potential to be both helpful and harmful, with effects varying with mood state. As part of the balancing act, individuals described having a strategic routine around their caffeine use to help manage their mood state and symptoms. Findings emphasise the importance of increasing knowledge surrounding caffeine use for both patients with BD and clinical professionals. Further research utilising both qualitative and quantitative methodologies to explore the caffeine‐affect relationship is warranted.

## Author Contributions

S.E., K.W. and J.T.G. were involved in the idea generation, design and methodology of the study. S.E. collected all the data, and S.E. and M.S. transcribed the interviews and undertook the data analysis. K.W. and S.D. provided guidance on data analysis methods and provided quality and credibility checking. S.E., M.S. and K.W. led the writing on the manuscript. All authors significantly contributed to the manuscript drafts and gave their final approval for publication.

## Funding

This work was supported by the Economic and Social Research Council, ES/P000630/1.

## Conflicts of Interest

K.W. receives occasional payment from clinical and academic organisations for workshops on psychological therapies for bipolar disorder. J.T.G. has received research funding from BBSRC, MRC, British Heart Foundation, Clasado Biosciences, Lucozade Ribena Suntory, ARLA Foods Ingredients, Cosun Nutrition Center, Innocent Drinks and the Fruit Juice Science Centre; is a scientific advisory board member to ZOE; and has completed paid consultancy for 6d Sports Nutrition, Science in Sport, The Dairy Council, PepsiCo, Violicom Medical, Tour Racing Ltd., and SVGC. For a full list of disclosures see https://sites.google.com/view/declarationsgonzalez/home.

## Supporting information


**Table S1:** A description of the ‘balancing act’ superordinate theme alongside the number of participants contributing to the theme and accompanying exemplar quote.
**Table S2:** Themes, subthemes and accompanying exemplar quotes describing ‘Low Mood’ and ‘Rock Bottom Low’ in the balancing act of the caffeine‐bipolar relationship.
**Table S3:** Themes, subthemes and accompanying exemplar quotes describing ‘High Mood’ and ‘Beyond the Realms of Reality and Reason’ in the balancing act of the caffeine‐bipolar relationship.
**Table S4:** Themes, subthemes and accompanying exemplar quotes describing ‘Habit/Routine’ and ‘Strategic Routine’ in the balancing act of the caffeine‐bipolar relationship.
**Table S5:** Themes and accompanying exemplar quotes describing ‘sleep’ and ‘physical activity’ as additional important components of the ‘balancing act’ of the caffeine‐bipolar relationship.

## Data Availability

Due to the sensitive nature of questions asked in this study, participants were assured their interview would remain confidential and that only key quotes from the transcripts would be shared in which their identity would remain anonymous.
