# Supplementary material for: ‘It's a Balancing Act’: A Qualitative Analysis of the Experiences of Caffeine Use in Individuals With Bipolar Disorder
Source: Bipolar Disord. 2026 Sep 30;28(7):e70171. doi: 10.1111/bdi.70171 (PMC13627961; doi:10.1111/bdi.70171)
Supplement: Supplementary file 1 [file BDI-28-0-s001.docx]

**Supplementary Material**

**Table S1**

*A description of the ‘balancing act’ superordinate theme alongside the number of participants contributing to the theme and accompanying exemplar quote*

| Superordinate Theme | Theme description | Number of participants contributing to theme | Exemplar Quotes |
| --- | --- | --- | --- |
| Balancing Act | Participants contributing to this theme described the ‘balancing’ involved in having bipolar and managing their mood symptoms. For many, caffeine had a very obvious and direct impact on their mood and therefore was important in the management and ‘balancing’ of the condition. Some participants actively described and talked about engaging in this balancing act. Many individuals describe caffeine to have a direct impact on their mood and bipolar symptoms and aspects of this balancing act, using caffeine strategically and the potential for caffeine to be helpful and harmful in relation to various mood states, yet were unaware they were engaging in this ‘balancing act’ process. | 18 | *“It's a balancing act is what it is. It's about not doing too much of anything… monitoring and managing mood.”* [Elizabeth]  *“I do believe that it's also for me an integral part because just in the mornings having that cup of calmness, cup of ... just getting the day started with a cup of coffee is definitely - for me - part of my treatment plan.”* [Ryan]  *“To me it [caffeine] is the most non-invasive product I can use to help me balance all those other things...To me this [caffeine] is almost the pillar that carries the rest of life. So if you take, so if this gets taken away, I really struggle to keep all the other bits going. This is the missing link.”*  [Karen] |

**Table S2.**

*Themes, subthemes and accompanying exemplar quotes describing ‘Low Mood’ and ‘Rock Bottom Low' in the balancing act of the caffeine-bipolar relationship*

| Theme | Subtheme | Theme description | Number of participants contributing to theme | Exemplar Quotes |
| --- | --- | --- | --- | --- |
| Little Helper |  | Participants described how caffeine could be very helpful in relation to low mood, lifting their mood, helping them to feel better | 17 | *“The caffeine is a little helper, it's a little side-kick to my tablets”. [Penny]*  *“On a low it makes you feel better, it lifts you up a bit”* [Elizabeth] |
|  | Crutch | Participants described being reliant on caffeine as a support in relation to lower mood, as a ‘pick me up’, helping them to cope and function with basic and necessary everyday activities. Some participants referred to caffeine as being an ‘essential medicine’. | 14 | *“extra boost… It's my crutch”* [Karen]  *“It's more a case of just taking that caffeine, you know, drinking that caffeine, just to sort of stay awake, and try, and try and sort of sharpen senses and things, so that I could maybe concentrate on a TV programme, or, or read a book, so in that sense, you know, it's more of an essential medicine I suppose”.* [Eric] |
|  | Comforting | Participants described caffeine being helpful in relation to low mood, having a comforting effect, being like a friend, providing a sense of calm, offering some reassurance and normality, and making them feel better. | 9 | *“Do you know what, it somehow calms me down, it gives me the reassurance, the feeling of you are going to be ok to have this extra bit of help in your life… it's my friend, it calms me down, it holds my hand”.* [Karen]  *“When I'm down, if the mood is a bit lower coffee has an even more comforting effect on me and it could also then result in me having a bit more coffee, almost seeking that cup of - I'd call it a cup of calmness or a cup of relaxation”.* [Ryan] |
| Nothing works |  | Participants talked about how when they are at ‘rock bottom’ low (the lowest of the low) that caffeine does not have the same effect. It does not seem to assist in helping them to manage their mood and to improve symptoms of depression. | 7 | *“There's a point of no return. There's a point you go beyond that it would be helpful”.* [Andrew]  *“To me it doesn't make any difference to how I feel when I drink coffee when I'm low. If I'm suicidal, I'm suicidal and a cup of coffee doesn’t change that”.*  [Katherine] |

**Table S3.**

*Themes, subthemes and accompanying exemplar quotes describing ‘High Mood’ and ‘Beyond the Realms of Reality and Reason’ in the balancing act of the caffeine-bipolar relationship.*

| Theme | Subtheme | Theme description | Number of participants contributing to theme | Exemplar Quotes |
| --- | --- | --- | --- | --- |
| Increased Manic-ness |  | Participants in this theme described how caffeine added to the high, exacerbating manic symptoms with the potential to lead to excessive consumption and this ‘upward spiral’ and chaining of behaviours. | 16 | *“When I'm high drinking a lot of tea can sort of wire me and sort of increase my 'manic-ness’.”*. [Victoria]  *“if I am in a higher state and I drank 2-3 coffees, I will be really flying around all day”.*  [Penny] |
|  | Reverse of Helpful | Participants described how caffeine’s ability to exacerbate manic symptoms in periods of higher mood as detrimental and harmful. | 13 | *“What you don't want to do is elevate what you're feeling when you're already high enough because that way lies madness …In a high mood, it [caffeine] might elevate me too much and there's a risk that I won't sleep, there's a risk that it pushed beyond what is useful for me and that's really unhelpful”.* [Elizabeth]  *“It's not going to be very helpful, is it? Not going to be helpful at all. It's going to be the reverse of helpful”.* [Andrew] |
|  | Complements Mania | Participants described how caffeine increased productivity and fuelled a state of mania and the impulsivity that it brings but did not describe this in a negative unhelpful or harmful way. | 8 | *“It keeps me focused on my work, the energy is more directed… it just keeps me focused.”.*  [Luke]  *“I have possibly drunk more coffee because I felt I need it to fuel my state of mania.”.* [Owen] |
| Can’t Recall/ Concentrate |  | Participants often referred to being unable to recall the caffeine consumed and the effects it had in relation to very extreme periods of high mood and/or unable to recall periods of high mood altogether, in addition, their mood was so high they could not maintain concentration on a single task for long enough to make and consume a caffeinated beverage. | 12 | *“Sometimes I think when you're really high you don't even take time to make yourself a coffee because you're dashing from one thing to another…you might put the kettle on and then you go off and do something else and then you haven't made the cup of coffee”.*  [Ester]  *“It's harder to reflect on that because everything's - everything goes so fast…you just going from one thing to another you know a bit like a butterfly really”.*  [Daniel] |

**Table S4**

*Themes, subthemes and accompanying exemplar quotes describing ‘Habit/Routine’ and ‘Strategic Routine’ in the balancing act of the caffeine-bipolar relationship.*

| Theme | Subtheme | Theme description | Number of participants contributing to theme | Exemplar Quotes |
| --- | --- | --- | --- | --- |
| Habit/Routine |  | Participants described a routine or habitual pattern of caffeine consumption. The habitual behaviour was most common and consistent when they are in ‘middle ground’. | 16 | *“It's kind of just out of habit really more than anything… sometimes I don't even feel like one and I just have it”.*  [Hannah]  *“I think it's one of those things that is more of a routine that I have”.* [Jack] |
| Strategic Routine |  | Participants contributing to this theme described having a strategic routine in relation to caffeine that involved deliberate actions that were important in the management of their mood and bipolar symptoms. | 17 | *“I use caffeine more as a tool in my repertoire rather than something I particularly like… I've got it in the toolbox, I use it when I need to and I carefully don't use it when I need not too. So I use my coffee allowances mindfully”.* [Elizabeth]  *“if you're Bipolar going into depression then strategic use of caffeine could be useful to give you a bit of a boost, a bit of a kick then”.* [Andrew] |
|  | More Caffeine | Participants described being reliant on and purposefully and deliberately consuming more caffeine in low mood as a way to help them to function and or due to the comforting effects it provided. | 7 | *“I use much more coffee in the low mood because I need the rocket fuel then”.* [Elizabeth]  *“If I’m feeling very low I will, you know, I’ll drink more coffee, I’ll want to, you know, do anything to try to feel better”.* [Katrina] |
|  | Self-care | Participants describe deliberately and strategically using caffeine to take care of themselves. This includes using caffeine in a calming, relaxing and mindful way in relation to both low and high mood and using caffeine to counteract effects of medication they take for their condition. | 9 | *“I associate coffee with giving me a space...  I think with mindfulness, it gives you the opportunity also to be more mindful about your situation. It gives me some help, if you are making a cup of coffee, there is a bit of a ritual in it isn’t there, that, it definitely gives you a chance for your mind to settle a bit”.* [Olivia]  *“In a way the coffee, I think I drink is to counteract some of the side effects of my prescribed medication, the sedative effect of that”.* [Eric] |
|  | Restriction of caffeine | Participants described restricting the amount of caffeine or certain types of caffeine, and or actively avoiding caffeine. This was predominantly in relation to high mood states, but some participants restricted and or avoided caffeine altogether. | 17 | *“I try to use sort of herbal teas and things like that, in some ways I try to avoid too much caffeine because I just know it’s going to make the manic mood worse really”.*  [Emma]  *“If I drink too much you know coffee during the day my minds starts working like I almost go into a hypomanic phase, so I have to be very mindful of that”.* [Katrina] |

**Sleep and physical activity**

Lifestyle behaviours ‘sleep’ and ‘physical activity where also described by participants to be important parts of the caffeine-bipolar ‘balancing act’, strongly connected to their ‘strategic routine’. Refer to Table S5 below for a breakdown of these themes. Sleep emerged as a very integral aspect in the management of bipolar episodes and mood symptoms; *“One way to prevent any further episodes* [hypomania] *is to be very careful on the amount of sleep I get per night... I know my mood changes anyway if I don't have enough sleep…”* [Anna]. Some participants described sleep to be a trigger or warning sign for both low and high mood states *“Sleep is the first sign of both my swings… whichever way my mood's going it pushes it further down that road”* [Elizabeth]. Participants collectively described negative implications caffeine directly had and or would potentially have on their sleep and how sleep is of vital importance in the management of bipolar. *“I do not like to overdo it because it will affect almost definitely my sleep pattern later on, you know going to sleep for Bipolar it's literally like the life and death importance.”*  [Penny]. This was particularly apparent in relation to periods of higher mood, which often resulted in sleep disturbances and reducing the need for sleep, with caffeine only amplifying these effects further. “*If I, I'm in a phase* [higher mood] *where I don't think I'm sleeping very... I might think the next day that I probably wouldn't have so much… Because I'm wanting to keep well and I'm very aware of sort of my mood and how you know I try to keep well and I wouldn't want to choose to take too much caffeine in case, in case it made me irritable or not sleep so well… it might make me a bit hyped up... it would be detrimental to my mood… cause me you know not to sleep”* [Ester]. One participant described how increased impulsivity during periods of high mood made it challenging to keep to their caffeine allowance as a voice of reason is not there. This often resulted in increased caffeine consumption, which could further affect sleep during a period of high mood; *“Because I think there is this filter that I have that sometimes I think I shouldn't have too much coffee because it might affect my sleep, but this filter would have gone. This reasonable voice in my head would have not been there”* [Anna].

Sleep was most commonly association with the detrimental effect it had to the onset episodes of ‘higher mood’. *“If I'm in a higher state already, my brain is pretty sharp, my brain's moving fast, I've got a lot of energy and so adding coffee into that mix would elevate it again. And it would mean that I'm less likely to sleep, which means my rhythms won't get back into middle ground as quickly and so that's why I avoid it so much when I'm in that headspace because it just continues the drive.”* [Elizabeth]. In addition, the ‘sleep’ theme links closely to ‘strategic routine’, specifically the ‘restriction of caffeine subtheme’, as participants’ described a strategic routine specifically and directly influenced by caffeine’s effects or potential to effect sleep that often resulted in the avoidance and or restriction of caffeine. *“If the sleep is being interrupted for a few nights in a row, I will try and cut it out and stick to lemon water until I get a solid sleep because like I said, sleep is paramount”*. [Penny]. *“I make sure I don't have caffeine before going to bed… Because I'm worried it's going to affect my sleep… I try and avoid anything that might affect my sleep”.* [Hannah] Participants described this strategic restriction of caffeine in relation to sleep which was very important for bipolar wellbeing. *“If I have caffeine sort of later on in the day, it basically makes it harder for me to fall asleep… I think my mood reacts to you know whether I've had enough sleep that night…I think it's a trigger… the times I have had mental issues, it always follows times when I've not been sleeping well.”* [Jack]

**Table S5**

*Themes and accompanying exemplar quotes describing ‘sleep’ and ‘physical activity’ as additional important components of the ‘balancing act’ of the caffeine-bipolar relationship*

| Theme | Theme description | Number of participants contributing to theme | Exemplar Quotes |
| --- | --- | --- | --- |
| Sleep | Participants talked about the importance of sleep in the management of mood symptoms and bipolar in general and the negative effects and implications caffeine had or could potentially have in relation to their sleep. Many participants spoke of deliberately and strategically avoiding consumption due to potential or experienced negative implications. | 12 | *“if the sleep is being interrupted for a few nights in a row, I will try and cut it [caffeine] out and stick to lemon water until I get a solid sleep because like I said, sleep is paramount, medication is paramount, food and drink is secondary.”* [Penny]  *“I won't have more than one [coffee a day]. It impacts on my sleep too much if I have more than one, that's a risk I can never take”.* [Elizabeth] |
| Physical Activity | Participants talked about the role of physical activity to be an important part of the balancing act to manage bipolar mood symptoms. This was most evident in relation to periods of lower mood. | 9 | *“it's all part of the balancing… I can't just take an exercise regime off the peg and use it… In the same way I do with coffee - where am I now? What effect would that exercise have?... I have to select the one [activity] that is necessary for that point… it's about the investment of energy…the aim of exercise is not to elevate my mood but to moderate it… it will lift or will bring my mood back to centre, whichever way I'm at.”* [Elizabeth]  *“If I want to have like a - I don't like to use the word normal life, but in the sense of not being in a manic phase and not being in a depressed phase - I can't see achieving that with treatment alone, in the sense of medication alone. You need to have your physical activity.”*  [Ryan] |
|  |  |  |  |

*Note. Participants experiences of physical activity were discussed in greater depth in a subsequent part of the interview and so will be reported in a future paper.*
